# Differential Diagnosis of Acute Phthisis and Typhoid Fever

**Published:** 1871-08

**Authors:** Levin J. Woollen


### *Differential Diagnosis of Acute Phthisis and Typhoid Fever.*
By Levin J. Woollen, M.D.

To diagnose betwen acute phthisis and typhoid fever is in many cases exceedingly difficult. There are few physicians long in practice who have not been brought face to face with this difficulty, and earnestly desired more light upon the perplexing subject.

The most familiar works on the practice of medicine do not give this differential diagnosis the consideration which its importance demands. Niemeyer states that in acute miliary tuberculosis the symptoms are "so very like those of typhus that the most experienced diagnosticians acknowledge to having met with instances in which a diagnosis between the two was absolutely impossible, and where patients dying with a diagnosis of typhus really had died of tuberculosis, and conversely." Yet Niemeyer's rules for the diagnosis are very unsatisfactory when applied to these diseases, at least as they occur in this part of the country.

Dr. Flint says that in making a diagnosis typhoid fever is to be excluded by the absence of the characteristic symptoms which belong to the clinical history of that disease; but unfortunately in acute phthisis we have many of the more prominent symptoms of typhoid fever.

Da Costa states that acute phthisis "often begins with a chill; fever follows; at first, like any inflammatory fever, with thirst, anorexia, quickened pulse, parched lips, and hot skin; but soon accompanied by exhausting night sweats and rapid emaciation, which, in connection with the intense restlessness and prostration and the frequent supervention of delirium, may cause the febrile disturbance closely to resemble typhoid fever." Again, the same eminent diagnostician says: "Acute phthisis may simulate other affections besides those of the chest. It has at times the delirium and prostration, the dry tongue, and the bronchial *ralés* of typhoid fever. The diarrhœa and the abdominal symptoms are, however, wanting. Yet simultaneous deposition of tubercles in the intestine may cause these; and in this case the only mark of difference from typhoid fever is the absence of an eruption."

My own experience is, that the peculiar eruption of typhoid fever is observed in but few cases. I have been so often disappointed in my efforts to discover it that I now seldom look for it, endeavoring to base my diagnosis on other and more certain grounds. In the further study of the means of distinguishing between these disorders, it will be best to examine the prominent symptoms occurring in each, and endeavor to show to which the weight of each symptom belongs.

According to Niemeyer there is, as a rule, an earlier appearance of the cough and dyspnœa and a greater intensity in tuberculosis than in typhus. In exanthematic typhus there are early and violent bronchitic symptoms; but the eruption of this disorder is highly characteristic, while there is none in miliary tuberculosis.

My answer is, that in the Western States, at least, we have cases of typhoid pneumonia where the cough and dyspnœa are prominent symptoms from the beginning, the abdominal disorder succeeding in a few days.

The author just referred to then asserts that in almost all the cases of abdominal typhus a careful examination will reveal a few spots of roseola upon the upper region of the abdomen. But, as I have already stated, the characteristic eruption of typhoid fever is, according to my observation, rarely discovered.

The third point referred to by Niemeyer is, enlargement of the spleen, rarely found in acute miliary tuberculosis, and then but slight; is almost always found in abdominal typhus. In reply it may be stated, that the physician would not feel himself justified in resting his diagnosis upon such evidence of an enlarged spleen as he could obtain; for did he believe, for example, that his case was one of acute phthisis, he would be very liable to attribute the increased dullness from such enlargement to tuberculous deposition, or other pathological change in the lung itself; and, on the other hand, were the case typhoid fever, intestinal tympanites would seriously interfere with the requisite examination below the false ribs. Besides, it has been justly observed that the spleen is sometimes found greatly enlarged without any apparent connection with other affections. "The enlargement may be of short duration; and it is then probably due, mainly or exclusively, to an accumulation of blood or engorgement."

While, according to Niemeyer, meteorism, liquid stools, and tenderness in the ileo-cœcal region—symptoms absent in acute miliary tuberculosis—are almost always present in abdominal typhus; yet, according to Da Costa, they may occur also in the former affection from a simultaneous deposition of tubercles in the intestines.

Niemeyer's fifth point is, that typhus rarely supervenes upon chronic disease of the lungs, while the other seldom attacks others than those thus suffering, and hence dullness at the apex of either lung is of great diagnostic significance.

Yet, in our country, typhoid fever does sometimes attack those who have chronic pulmonary disease, or such as have, to use their own words, "weak lungs." These cases, however, have a less fatality than those occurring in persons previously robust and healthy, especially just after adolescence. Marked dullness at the apex of either lung will seldom be found; "for the tubercles being minute and discrete, and moreover existing equally in both lungs, exploration of the chest may furnish no marked disparity in the resonance on percussion, and none of those modifications of the respiration and voice which denote pulmonary solidification."

Finally, Niemeyer states, upon the authority of Wunderlich, that the temperature is much lower in acute miliary tuberculosis than in typhus, seldom reaching 104° F., and is out of all proportion to the great rapidity of the pulse.

The evidence derived from temperature can only be corroborative; it is not sufficient to determine a doubtful diagnosis. And, moreover, few physicians are provided with thermometers, or at any rate are in the habit of making thermometric observations, at least in country practice.

Having thus examined these points as presented by Niemeyer, and found them by no means conclusive, the inquiry still remains, How are the two diseases to be distinguished? It must be acknowledged that no *entirely* satisfactory answer can be given. From my own experience, somewhat limited it is true, I would say, first, that the *facial expression* will be of as great value as any one symptom.

Thus the patient has a peculiar look or expression in severe cases of typhoid fever (and these are the ones we are liable to confound with phthisis) not found in any other disease. Of course it is not possible to convey in words an intelligent description of such expression. It is something to be learned at the bedside, and there only. "The countenance is marked by great debility and tremulousness of the muscles, and by great sinking. The bones are more prominent, the intervening spaces more sunk and depressed than natural. The surface is sometimes slightly flushed, and sometimes cool and clammy. The eye-lids are frequently partly closed, and the eyes suffused, dull, and covered with a film of mucus. The mouth is apt to be partly opened; the teeth and lips affected with dark-colored glutinous sordes. The articulation is difficult and imperfect, and attended with great effort, and with tremor and an inadequate action of the lips and of the tongue, which is put out with tremor and difficulty."

Next, in those cases of acute phthisis that have come under my own observation, in which the delirium appeared early, I found it more *active* than the ordinary *typhomania*. Thus in one case, where acute phthisis supervened upon rubeola, the patient during most of her waking hours for a few days was engaged in singing hymns—many of them being improvised.

The dull eye and half closed lids of typhoid fever were absent, and *her mind dwelt on things around her*, in this respect differing from typhoid fever, in which disease, as Watson says, the mind is elsewhere. "The patient wanders at first in the night only, and the delirium commonly appears on his waking from disturbed sleep. Sometimes he can only be kept in bed by the imposition of some restraint. Usually, however, his rambling is of a tranquil kind, and without agitation. *His mind seems elsewhere.* He is inattentive to all that passes around him; but he lies still, muttering disjointed words or sentences, like a man talking in his dreams."

*The evidence afforded by the tongue* is entitled to some though no great weight. In those cases of acute phthisis to which my attention has been called, the tongue, if it became dry, pursued a different course in attaining its dry condition from the *ordinary* dry tongue of typhoid fever. Thus, I believe, without exception, I noticed that the organ was covered with a white fur, which became thicker and more prominent until it "slipped off" hurriedly, as it were; and, instead of leaving the surface in anything like a normal condition, it was found to be *red, glassy and dry.* In ordinary cases of typhoid fever the tongue, being covered with a white coat, gradually changes to a brown; and this brown coat, becoming more prominent, even shows signs of dryness; the dryness in most cases beginning at the tip and in the center, and gradually extending over the whole upper surface of the organ. I am aware, however, that in some cases of typhoid fever the coating falls off suddenly, and leaves the surface red, glassy and dry. In such cases I think I could trace, if not tubercular deposit in the intestines, at least a marked tubercular diathesis; and the patients, if they recover from the existing disease, will, as a rule, ultimately fall victims to phthisis.—*American Practitioner.*

